# Auxin Control of Root Organogenesis from Callus in Tissue Culture

**DOI:** 10.3389/fpls.2017.01385

**Published:** 2017-08-08

**Authors:** Jie Yu, Wu Liu, Jie Liu, Peng Qin, Lin Xu

**Affiliations:** ^1^National Key Laboratory of Plant Molecular Genetics, CAS Center for Excellence in Molecular Plant Sciences, Institute of Plant Physiology and Ecology, Shanghai Institutes for Biological Sciences, Chinese Academy of Sciences Shanghai, China; ^2^University of Chinese Academy of Sciences Beijing, China; ^3^Department of Instrument Science and Engineering, Shanghai Jiao Tong University Shanghai, China

**Keywords:** plant regeneration, tissue culture, callus, adventitious root, *de novo* root regeneration

## Introduction to direct and indirect *de novo* root regeneration

**Figure 1:**
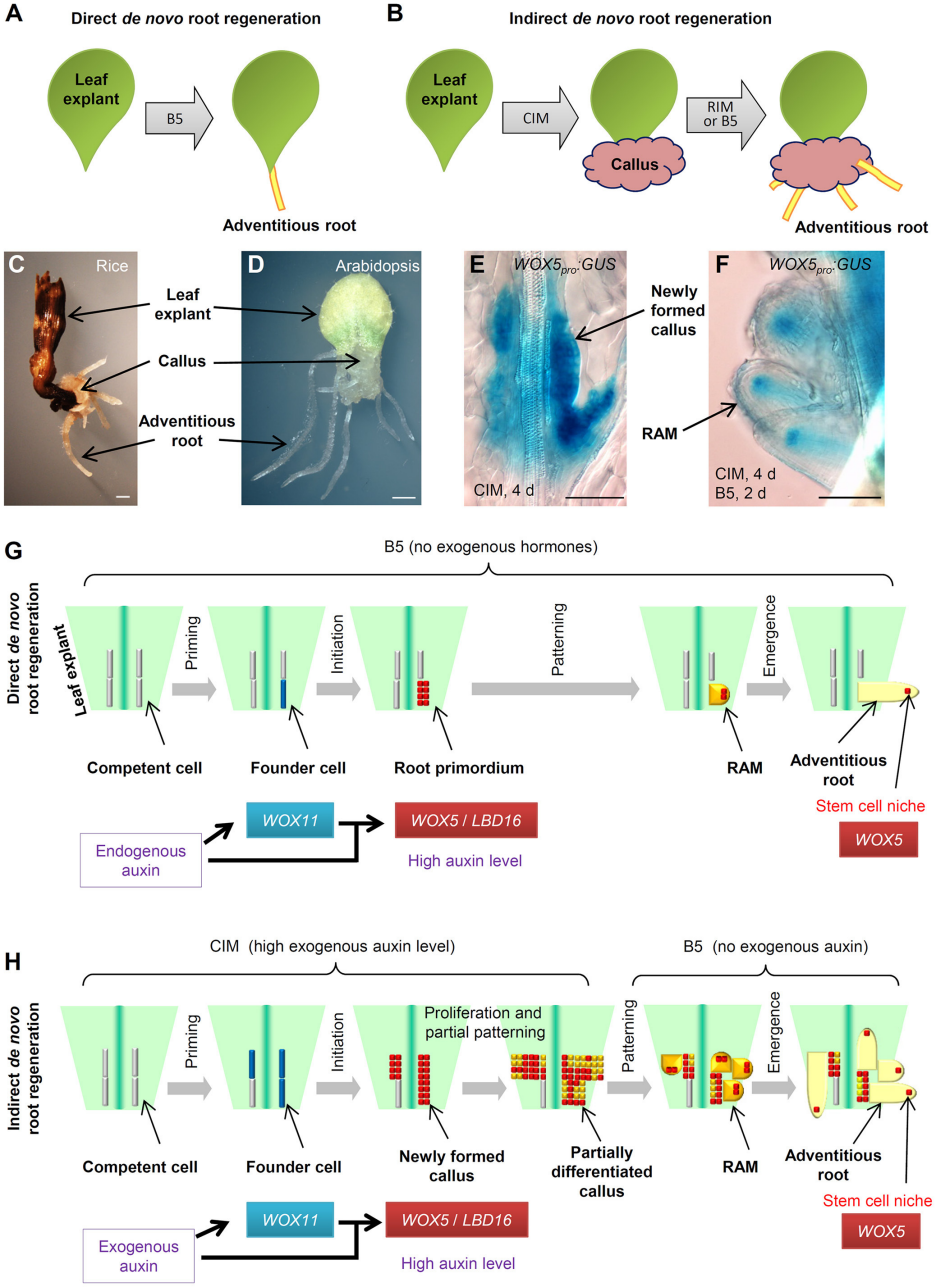
Direct and indirect DNRR. **(A)** A system to study direct DNRR. Leaf explants were cultured on B5 medium without added hormones (Chen et al., 2014). **(B)** A system to study indirect DNRR. Leaf explants were cultured on CIM to induce callus and were then transferred to RIM or B5 medium to produce roots. **(C,D)** Indirect DNRR from rice **(C)** and Arabidopsis **(D)**. Leaf explants were first cultured on CIM for 11 d **(C)** or 6 d **(D)** and then transferred to B5 medium for another 6 d **(C)** or 5 d **(D)**. **(E,F)** *WOX5*_*pro*_*:GUS* (He et al., 2012) in callus on CIM **(E)** and in roots after transferred to B5 medium **(F)** during indirect DNRR. Leaf explants were first cultured on CIM for 4 d before being transferred to B5 medium for another 2 d. Notably, the GUS signal was strong in newly formed callus cells on CIM **(E)** and was gradually restricted to the stem cell niche in root tips after transferred to B5 medium **(F)**. **(G,H)** Proposed cell lineage in direct DNRR **(G)** and indirect DNRR **(H)**. Scale bars, 1 mm in **(C,D)** and 100 μm in **(E,F)**.

During post-embryonic development, roots can be initiated by a programmed developmental order or by environmental and wound stimulation (Bellini et al., 2014; Xu and Huang, 2014; Birnbaum, 2016; Ikeuchi et al., 2016; Kareem et al., 2016; Lup et al., 2016; Rellan-Alvarez et al., 2016; Steffens and Rasmussen, 2016). *De novo* root regeneration (DNRR) is a type of plant regeneration to produce adventitious roots upon wounding or stress (Liu et al., 2014; Xu and Huang, 2014). For example, using leaf explants of Arabidopsis (*Arabidopsis thaliana*), adventitious roots could usually be regenerated by two ways: adventitious roots could be formed directly from detached leaf explants when cultured on B5 medium without added hormones (Chen et al., 2014; Liu et al., 2014), hereafter called direct DNRR (Figure 1A); or adventitious roots could be formed from callus in tissue culture, hereafter called indirect DNRR. In indirect DNRR, leaf explants are first cultured on callus-inducing medium (CIM) with high auxin levels to induce callus formation, and then the callus is transferred to root-inducing medium (RIM) with low auxin levels or even on B5 medium without auxin supplement to allow root formation (Figure 1B).

Many plants such as Arabidopsis and rice (*Oryza sativa*) can form adventitious roots from callus (Figures 1C,D). While the cell fate transition during direct DNRR from Arabidopsis leaf explants has been carefully studied in Arabidopsis (Liu et al., 2014; Chen et al., 2016b,c; Hu and Xu, 2016; Sheng et al., 2017), in indirect DNRR the cell fate transition is still not clear. How roots are formed from callus remains unanswered. In this paper, we present our analyses of the cell lineage of indirect DNRR and discuss the similarities and differences between direct and indirect DNRR.

## Cell fate transition during direct DNRR

Here, we summarize the four steps of cell fate transition involved in direct DNRR from leaf explants together with adventitious rooting in other systems (Figure 1G). In the first step “priming,” the endogenous auxin is transported into regeneration-competent cells (i.e., procambium and vascular parenchyma cells) in the vasculature near the wound and activates *WUSCHEL-RELATED HOMEOBOX11* (*WOX11*) expression for the fate transition from regeneration-competent cells to root founder cells (Liu et al., 2014; Chen et al., 2016a,b). In the second step “initiation,” *WOX11* and auxin coordinately activate *WOX5* and *LATERAL ORGAN BOUNDARIES DOMAIN16* (*LBD16*) expression for the fate transition from root founder cells to root primordium (Liu et al., 2014; Hu and Xu, 2016; Sheng et al., 2017). *WOX11* expression then decreases in this step (Liu et al., 2014; Hu and Xu, 2016). Auxin keeps a high level in the root primordium. In the third step “patterning,” cell division continues in the root primordium, which begins to differentiate into a root apical meristem (RAM). The auxin level is tuned down and auxin distribution is restricted to the tip of the meristem to confine the region of the stem cell niche (De Klerk et al., 1999; Della Rovere et al., 2013; Druege et al., 2016). *WOX5* is gradually restricted into the stem cell niche and *LBD16* expression decreases (Hu and Xu, 2016). In the fourth step “emergence,” the mature root tip and stem cell niche are formed and the root tip grows out of the leaf explant (Chen et al., 2016c; Hu and Xu, 2016).

## Cell fate transition during indirect DNRR

In tissue culture, adventitious roots could be obtained via indirect DNRR (Figure 1H). On CIM, callus is induced from leaf explants by a high level of auxin. Recent theory suggests that callus formation is via the rooting pathway (Che et al., 2007; Atta et al., 2009; Sugimoto et al., 2010; Fan et al., 2012; He et al., 2012; Liu et al., 2014) and also involves two cell fate transition steps in Arabidopsis (Liu et al., 2014). In the first step (the priming step) of cell fate transition from regeneration-competent cells to founder cells, *WOX11* is specifically induced in founder cells (Liu et al., 2014). In the second step (the initiation step) of cell fate transition from founder cells to callus, *WOX11* expression decreases while *WOX5* expression increases in the newly formed callus (Liu et al., 2014; Figure 1E). *LBD16* expression is also observed in the newly formed callus (Fan et al., 2012). Therefore, the newly formed callus seems to be a group of root primordium-like cells that is under the control of the high auxin level from CIM (see “newly formed callus” in Figure 1H).

Ideally, under continuous stimulation with a high auxin level, the status of callus is maintained at the root primordium-like status. However, in tissue culture, auxin might not be evenly distributed in the callus mass and there is always partial differentiation of callus as some callus cells try to enter the patterning step. Many root meristem genes were observed in diverse domains of the fast dividing and partially differentiated callus mass (Sugimoto et al., 2010; Kareem et al., 2015). *WOX5* and *LBD16* may not be ubiquitously expressed in the partially differentiated callus. Therefore, the partially differentiated callus could be at any stage from root primordium to root meristem and could be composed of many different types of meristem cells with diverse gene expression patterns (see “partially differentiated callus” in Figure 1H). We believe that this is a balanced result from the tug of war between the exogenous auxin stimulation and the endogenous developmental program. On one side, the high level of exogenous auxin attempts to maintain the callus at the root primordium-like status because the root primordium has a high auxin level (Sabatini et al., 1999; Benkova et al., 2003; Okumura et al., 2013; Liu et al., 2014) and consists of precursor cells of the stem cell niche (Hu and Xu, 2016). On the other side, the endogenous developmental program tries to force this group of root primordium-like callus cells into the patterning step to differentiate into the RAM. As a result of the balance of these two forces, the callus mass maintains some of the root primordium features while there is also partial differentiation with some RAM traits.

When callus is moved to RIM or B5 medium, the removal of auxin in the medium results in the loss of the ability to keep the callus at the root primordium-like status. The endogenous developmental program then drives the callus to finish the patterning step during which *WOX5* is gradually restricted to the stem cell niche of newly formed RAMs (Figure 1F). As there are plenty of root primordium-like cells present during callus formation, we could observe the formation of numerous adventitious root tips from the callus on RIM or B5 medium.

## Comparison of the two rooting types

Here, we summarize the similarities and differences between the two types of adventitious rooting from leaf explants (see models in Figures 1G,H). Based on the above hypothesis, we propose that direct DNRR and indirect DNRR have very similar cell fate transition steps. They all experience priming, initiation, patterning, and emergence steps to finally form adventitious roots. Additionally, the molecular markers are also similar, involving *WOX11* for founder cells and *WOX5*/*LBD16* for root primordium and newly formed callus. *WOX11* is generally involved in adventitious root formation and callus formation in both Arabidopsis and rice (Zhao et al., 2009; Liu et al., 2014; Hu et al., in press).

There are two major differences between direct DNRR and indirect DNRR. First, the auxin source for the two types of rooting is different. In direct DNRR, endogenous auxin is mainly produced in mesophyll cells, leaf margin cells and some other cells in the leaf explant and then transported into regeneration-competent cells (Liu et al., 2014; Chen et al., 2016b), while in indirect DNRR, exogenous auxin is mainly provided from the medium. Therefore, mesophyll and many other cells in the leaf explant are required for direct DNRR (Chen et al., 2016a,b) but may not be required for indirect DNRR.

Second, the auxin behavior is different in the two types of rooting. In direct DNRR, the cell fate transition is strictly controlled by endogenous auxin and developmental programs. The auxin concentration is focused just in a few cells (the root primordium) in direct DNRR, and therefore the regenerated root number is limited (usually 1–3 roots). The endogenous auxin keeps a high level in the root primordium and its level decreases during patterning. The rooting process usually does not stop between initiation and patterning. However, during indirect DNRR, the priming and initiation steps of cell fate transition are quite dramatic and fast, and numerous regeneration-competent cells are induced to form founder cells that then divide to become newly formed callus following stimulation with high levels of exogenous auxin. Auxin is enriched in many callus cells, and therefore numerous adventitious roots could be observed when auxin is removed in indirect DNRR. In the newly formed callus, the high level of exogenous auxin in CIM prevents callus from patterning, resulting in disruption of the recognition of the tissue anatomy and keeping callus cells without pre-specific fate. Removal of auxin from the medium allows callus into the patterning step.

Overall, we believe that direct DNRR and indirect DNRR share similar cell fate transition steps but have different auxin sources and behavior. Further studies on genetic and epigenetic regulations of direct and indirect DNRR will improve our understanding of the two ways of adventitious rooting at the molecular level. We cannot exclude the possibility that adventitious roots can also initiate from differentiated cells via cell fate reprogramming. Cell lineage analysis using *WOX11, WOX5, LBD16* and other markers and phenotype analysis of mutants are important in studying different types of rooting in the future.

## Author contributions

JY, WL, JL, PQ, and LX designed the research. JY, WL, and JL conducted the research. LX wrote the manuscript.

### Conflict of interest statement

The authors declare that the research was conducted in the absence of any commercial or financial relationships that could be construed as a potential conflict of interest.
